# Privacy versus open science

**DOI:** 10.3758/s13428-019-01259-5

**Published:** 2019-05-31

**Authors:** Simon Dennis, Paul Garrett, Hyungwook Yim, Jihun Hamm, Adam F. Osth, Vishnu Sreekumar, Ben Stone

**Affiliations:** 1 University of Melbourne, Melbourne, Victoria Australia; 2 University of Newcastle, Callaghan, New South Wales Australia; 3 University of Tasmania, Hobart, Tasmania Australia; 4 Ohio State University, Columbus, OH USA; 5 National Institutes of Health, Washington, DC USA; 6 Unforgettable Research Services Pty Ltd, Melbourne, Victoria Australia

**Keywords:** Privacy, Open science, Open repositories, Differential privacy

## Abstract

Pervasive internet and sensor technologies promise to revolutionize psychological science. However, the data collected using these technologies are often very personal—indeed, the value of the data is often directly related to how personal they are. At the same time, driven by the replication crisis, there is a sustained push to publish data to open repositories. These movements are in fundamental conflict. In this article, we propose a way to navigate this issue. We argue that there are significant advantages to be gained by ceding the ownership of data to the participants who generate the data. We then provide desiderata for a privacy-preserving platform. In particular, we suggest that researchers should use an interface to perform experiments and run analyses, rather than observing the stimuli themselves. We argue that this method not only improves privacy but will also encourage greater compliance with good research practices than is possible through open repositories.

The scientific community is in the midst of two revolutions that are about to collide. On the one hand, as a consequence of the replication crisis that has occurred across multiple disciplines (Open Science Collaboration, 2015), researchers are being encouraged to share their data in open repositories, thus facilitating reanalysis and increasing the transparency of the scientific enterprise. At the same time, the introduction of a range of consumer sensor devices, along with the availability of online social data, is providing researchers with an unprecedented window into the everyday lives of people. The data collected using these experience-sampling methodologies have the potential to transform our understanding of human behavior. However, it is an unfortunate fact that the most valuable data are often the most sensitive. Our ability to fully exploit these new data sources will be directly related to our ability to preserve the privacy of the people who provide the data.

The Cambridge Analytica scandal, in which tens of millions of Facebook profiles were leaked to a political consulting firm that used them to try to influence the 2016 US presidential election, demonstrated both the power and the danger involved in large-scale data collection. As the public takes stock and governments react, we can expect research practices to come under increasing scrutiny. There is a very real danger that the public good that could be derived from studying dense datasets will not be realized.

The Cambridge Analytica case is particularly instructive in that there was no “hack.” The data were given to a legitimate researcher, Aleksandr Kogan from Cambridge University, as he was conducting personality research on the platform. He then passed the data to Cambridge Analytica, allegedly in breach of Facebook’s terms and conditions.

The case illustrates a fundamental fact of data—once data are given, they are very hard to take back. Cambridge Analytica claims to have deleted all copies and to have submitted to audit, but there really is no way to put the genie back in the bottle with complete confidence. If we are to realize the full potential of big data without seriously compromising civil liberties, we need to reconceptualize our relationship to data.

In particular, one cannot simply upload participants’ email, Global Positioning System (GPS), phone call, and short message service (SMS) data to a public repository. Even if obvious personal identifiers such as names and addresses are removed, reverse-engineering identity from diverse sources is surprisingly easy (Narayanan & Shmatikov, 2010). To upload data knowing that one cannot protect the identity of participants is both unethical and ultimately self-defeating, since engaging participants will become very difficult once they understand the implications of being involved in these studies.

In this article, we will discuss the value of experience-sampling paradigms, the objectives of the open science movement, and provide a pathway to resolve the conflict by discussing the architecture of a privacy-preserving experience-sampling platform.

## The promise of experience sampling

Most empirical research in psychology either involves administering surveys across a cohort or occurs in the laboratory. Although a great deal has been learned about psychological processes using these methods, they provide very sparse samples of human behavior under quite unnatural conditions. Experience-sampling methods (Csikszentmihalyi & Larson, 1992) use smartphones, wearable sensors, social media, and the internet of things to collect much denser data over longer time periods, as people engage in the activities of daily living. For instance, in an ongoing project with bipolar patients, we are collecting accelerometry data 8–15 times a second, 24–7, for a year. These accumulate to about a half billion data points per participant, providing a fairly complete record of their movement during the collection period.

Experience-sampling methods (ESM) come in both passive and active forms. Passive ESM involves the use of data sources that people generate automatically as a consequence of their activity, such as accelerometry and GPS. Active ESM (also known as *ecological momentary assessment*; Shiffman, Stone, & Hufford, 2008) interrupt participants throughout their day and requires them to provide a response. Although active ESM are less naturalistic than passive ESM, they allow the sampling of internal mood and other cognitive states that cannot be reliably ascertained on the basis of passive data alone. We are using active ESM to measure mood states in the bipolar study mentioned above.

With the appropriate analysis methods, one can ask questions using ESM data that cannot be addressed using traditional methodologies. For instance, we have been able to characterize the dimensionality of people’s visual experience (Sreekumar, Dennis, Doxas, Zhuang, & Belkin, 2014), to examine the neural representations of time and space over time scales up to a month (Nielson, Smith, Sreekumar, Dennis, & Sederberg, 2015), and to model the processes involved in real-world episodic memory (Dennis et al., 2017).

The last of these studies illustrates the promise that ESM hold. In this study, participants wore a smartphone in a pouch around their necks for two weeks. The phone collected audio, image, movement, and spatial information. A week after data collection, the participants were presented with a selection of their images and asked on which day they had been taken. Because of the specificity of the data, predictions could be made on an item-by-item and person-by-person basis, and the relative contributions of audio, visual, movement, and spatial information to memory performance could be assessed.^1^

In the laboratory, it is common for experimenters studying episodic memory to administer lists of random words for participants to study, deliberately striping away the structure that participants might exploit in their everyday memory in order to better elucidate the underlying processes. However, when presented with an unfamiliar structure, the participants might adopt strategies that they do not normally employ. The introduction of control may perversely complicate the problem that researchers are trying to solve, as the results of such experiments might not reflect the type of retrieval that participants normally engage in.

Furthermore, people’s lives are dominated by repeating experiences (Sreekumar, Dennis, & Doxas, 2017; Sreekumar et al., 2014), and such recurrence structures are thought to influence performance (Dennis & Humphreys, 2001; Osth & Dennis, 2015). ESM provide a way to quantify recurrence structures in people’s lives and how they relate to cognition—something that has not been possible until now.

Across a range of areas, experience-sampling approaches promise to provide a more comprehensive, ecologically valid, and translationally relevant psychological science. However, the data being collected are very sensitive, and issues of ownership and access become much more acute. In the next section, we discuss the open science movement and highlight the fundamental conflict between it and experience-sampling research.

## The open science movement

Driven by the specter of the replication crisis (Open Science Collaboration, 2015), a deepening concern for the integrity of scientific evidence has led to the open science movement and the construction of best-practice guidelines for research (Nosek et al., 2015). *Open science* is a term that incorporates a set of distinct practices and perspectives (Mehler & Weiner, 2018; see in particular the Whitaker quote). *Open* can refer to:

- the transparency of research practices and analysis methods (with the rise of preregistration as an emerging standard for confirmatory research)
- the degree of access that people have to research (particularly taxpayer-funded research)—open access
- the transparency of the commercial and other motivations for conduct of the research
- the ability of people to examine, contribute to, and use software—open source
- the ability of nonacademics to engage in the scientific process—citizen science
- the ability of all people to enter academia, regardless of race, gender, nationality, and so forth
- the ability to access the data from which conclusions have been drawn—open data

Working toward open science in all of these senses is laudable. In this article, however, we are concerned with the problems associated with open access to data. An increasing number of repositories and sharing standards have been created to facilitate this aim. These include Dataverse (King, 2007), Dryad (White, Carrier, Thompson, Greenberg, & Scherle, 2008), the Inter-university Consortium for Political and Social Research (ICPSR; Taylor, 1985), the Open Science Framework (Foster & Deardorff, 2017), OpenNeuro (Poldrack & Gorgolewski, 2015), brainlife.io (Pestilli et al., 2019), the human connectome project (Van Essen et al., 2013), Biobank (Sudlow et al., 2015), and the Qualitative Data Repository (QDR; Kirilova & Karcher, 2017). Other commentators have advocated the immediate and automatic uploading of data to stores such as github—the so-called “born-open approach” (Rouder, 2016). In many laboratory paradigms, sharing in this fashion is unproblematic. When the data are more sensitive, we would argue that open sharing of this form can have negative consequences, and is illegal in many cases.

For instance, if you are collecting the emails of individuals, releasing them to an open repository may reveal information about the author’s relationships or activities that is personally compromising. Similarly, releasing people’s GPS locations at given times would allow thieves to discern when people will not be home, and therefore make the participants vulnerable. If participants’ financial documents, social security numbers, credit card details, and so forth are made publically available, they could be subject to identity theft. Releasing the results of surveys about people’s personalities or political views could be used to influence elections, and making their health records available might influence insurance or employment decisions inappropriately.

There are some circumstances under which open sharing might be justified. For instance, after the Enron scandal in 2001, a database of email exchanges between members of the company was released to the community for research purposes. In this case, it was decided that the value to society of releasing the database overrode the rights of the individuals involved. However, such cases are rare, and the degree of concern that has surrounded breaches such as the Cambridge Analytica case suggests that most people would not consider the open sharing of these kinds of sensitive information acceptable even when a case can be made for a public good.

The problems associated with sharing data are particularly acute in the case of qualitative data, so the issue has been a longstanding point of discussion (Kirilova & Karcher, 2017). Qualitative researchers tend to foster much closer relationships with their participants, and even before the advent of sophisticated computational techniques for reverse-engineering identity, the data they collected were difficult to deidentify. The nature of qualitative analysis requires researchers to personally engage with their raw materials. Many qualitative researchers have come to the conclusion that one should never share data. Others, however, have sought to elucidate policies and processes to allow sharing (Kirilova & Karcher, 2017).

A key issue raised by this work is the nature of consent. Kirilova and Karcher (2017) advocate that researchers engage in extended conversations and provide participants with multiple options in terms of the way their data might be shared. Such a policy, though, places a great deal of responsibility on the researcher to interpret the participants’ wishes. The willingness to share might depend on many factors—most notably, the purpose of the research—that are not available at the time the data are collected. The question arises of whether data should ever be shared without the consent of the participants in that particular case. Underlying this question is an even more fundamental one—to whom should the data belong?

## Who owns data?

A critical issue in the discussions of privacy is who should own data. In current practice, the ownership of the data that psychological researchers collect typically transfers to the researchers’ host institution. Restrictions to this ownership enshrined in ethical protocols and privacy law usually afford participants the right to amend and/or delete data that they have provided (e.g., the Australian Privacy Act 1988, www.oaic.gov.au/privacy-law/privacy-act/). In practice, logistical barriers tend to mean that few participants exercise their right to modify their data, and researchers typically treat data as if they own them—for instance, feeling little compunction about taking a dataset from one institution to the next when they move, and/or publishing to open platforms without seeking institutional approval.

Although participants may show little concern about giving away an hour’s worth of laboratory data, they may feel rather differently about giving away experience-sampling data that may have been collected over several years and contain much more sensitive information. The public outcry about Facebook and the Cambridge Analytica scandal demonstrates that individuals are becoming increasingly protective and concerned about their online data and the privacy of those data.

Any such discussion necessarily sits within the context of efforts by government to institute national data banks. In the health sphere, these efforts are gathering pace. In 2016, the Australian government launched “My Health Record,” a permanent electronic record of interactions with healthcare providers across the nation. On February 25, 2018, 5.5 million people were registered with My Health Record (23% of the Australian population), and some 10,754 healthcare providers were connected (Australian Digital Health Agency, 2018). The objective is to reach 98% coverage by the end of 2018. Similar efforts have been underway for some time across multiple countries (Ludwick & Doucette, 2009). Although the potential advantages are substantial, the government ownership of data remains controversial (Anderson, 2007; Gagnon et al., 2016). Similarly, the corporate ownership of data is coming under increasing scrutiny, due to events like the Cambridge Analytica breach discussed earlier.

An alternative to institutional ownership of data is to have participants retain ownership. Under this model, data become an asset that participants allow researchers to license—either in the interests of the public good or for compensation. Participants would build a personal data warehouse that might include generic data that could be used for multiple purposes (such as GPS coordinates) that could be combined with surveys or experimental responses requested by researchers. As time progresses, the value of the data asset would grow with its extent. Researchers might then offer compensation for a given type of data, and participants would consent on a case-by-case basis. The researchers would be purchasing the right to analyze data, not the data themselves, so the participant would then be free to participate in other studies (including replications) and to earn additional compensation from other researchers for the same data.

Although this proposal requires a shift in the way in which researchers understand their relationship to data, it has a number of advantages:

- Participants would make decisions about the use of their data on a case-by-case basis—a form of dynamic consent (Kaye et al., 2015; Williams et al., 2015). Researchers would provide a statement about the use to which the data would be put in their request, and participants would provide their consent with this use in mind. Ethics boards, advocacy groups, and government are also likely to play roles in deciding which projects are appropriate, but we would argue that in most cases participants should retain the right to control their data.
- Participants would be incentivized to curate their data to ensure the data are as complete as possible, as this would make the data more likely to be requested. Missing data is a much more significant problem in experience-sampling paradigms than is typically the case in laboratory work, so any dynamic that engages participants is desirable.
- Currently, people’s understanding of the relative value of data and the privacy implications of allowing others access is rudimentary. Global information technology companies like Google and Facebook collect large amounts of data in exchange for allowing people to access their systems, but they do not provide financial compensation. If participants retain ownership of their data and participate in a data marketplace, they will come to understand which kinds of data are most valuable, both to them collectively and to researchers. The promise then is that a more nuanced understanding of privacy would emerge.
- In many cases, the data that are most valuable to researchers belong to members of special populations who are commonly financially disadvantaged. Ensuring they are able to retain ownership of their data could provide a supplemental income to people with financial needs. If this mechanism is to work to a substantial degree, the data should be seen as capital for rent, not as labor, as is currently the case with internet work providers such as Amazon Mechanical Turk or Prolific Academic.
- The weak link with current open repositories is the time between publication of the article and posting of the data. Well-meaning researchers struggle to format, document, and post their data. Publication standards aimed at sharing will certainly affect this tendency; however, this will require surveillance and enforcement. By contrast, in the approach advocated herein, the data would be submitted directly to a repository by the participant. Using a key published with the article, a replicator could immediately access the set (with the permission of the participants) without additional processes, thus removing a key impediment to sharing (cf. born open; Rouder, 2016).
- Participant ownership of data could lead to increased engagement in and understanding of the scientific process—common objectives in citizen science projects (Bonney et al., 2014).

To realize this kind of privacy-preserving platform, several technical challenges must be addressed. We outline these in the next section.

## A privacy-preserving platform

There are several critical aspects to consider when designing a privacy-preserving experience-sampling platform. These include the collection mechanisms, the search and visualization interfaces, the data analysis platform, the experimental platform, and the legal framework in which the service operates. An (Australian) example of legal privacy standards can be found in the Appendix.

### Collection mechanisms

The first question when trying to maintain privacy is which data are to be collected in the first instance. Too often, current apps and services lack transparency in what they collect. Even when it is possible to select what forms of data are collected, the interface is often obscure. Apps and services can implement a few design principles to improve their privacy interfaces:

- There should be a prominent “all stop” button, to allow users to cease all recording when they wish and to easily resume collecting when they wish. If users must disable each data stream individually (e.g., GPS, audio, and accelerometry), there is a greater probability that they will miss one and continue collecting data when they did not intend to.
- Conversely, users should be able to turn on and off individual data streams so that they can decide what they are comfortable sharing, and they should be able to change this at any time. Each stream comes with a different trade-off in terms of the privacy that is relinquished and the degree to which relinquishing privacy is necessary for the purposes of collection. Excessive bundling can be used to coerce users to share data they would not otherwise choose to share.
- When data are collected on a smartphone or similar device, there ought to be a delay between when the data are collected and when they are uploaded, during which the user can delete the data—somewhat like the mechanisms that live television programs implement to avoid broadcasting inappropriate content. Once data leave the device, they are more difficult to control, so there should be an opportunity for users to prevent data from uploading.
- Consideration should be given to the format of the data being uploaded, to assess whether a more private form would serve similar purposes. For instance, when recording audio it may not be necessary (or legal) to retain raw audio in a form that can be replayed. Sometimes, however, it is enough to sample sporadically and retain only frequency information. Machine-learning algorithms can be used to determine ambient qualities of the audio, such as whether it contains voices or traffic, without being able to replay the stream. As another example, when recording phone calls or SMSs, for many research purposes it is sufficient to retain the time of calls and perhaps a unique identifier for the caller that is not the caller’s name or number. In this way, the temporal characteristics and diversity of callers can be ascertained without retaining more sensitive information, such as the content of SMSs or the identity of callers. It is a common maxim in experimental research that one should record everything, as you can never be sure what future analyses you might want to conduct. With private data, however, that edict must be balanced against the need to protect participants from future analyses they might not want conducted.

Knowing which data to retain and which not to is not trivial. A baseline position is to insist on adherence to the law. Legislation exists with respect to the collection of raw audio, although it differs by jurisdiction. In some states of the US, for instance, it is illegal to record conversation unless all parties captured agree (Justia, 2019). In many experience-sampling protocols, it is impractical to obtain permission from all passersby, so raw audio cannot legally be retained. For most data streams, however, explicit legal guidance is not available. The dominant approach currently is to rely on ethics boards to endorse only those projects that collect appropriate information. In our experience, the ethics boards at different institutions vary markedly in what they consider acceptable.

A second approach is to conduct studies to establish the social license from participants for data-gathering and analysis activities. We are currently conducting a study to determine the appropriate use cases for the wifi connection data that the University of Melbourne collects from people who visit the Parkville campus. The study will provide participants with triads of scenarios that specify multiple aspects of potential cases, including what will be collected, how data will be linked with other sources (e.g., email address databases), the purpose of the research, and what benefits will accrue, both to the person whose data are being collected and to society more generally. The participants will be asked to select the best and worst of these scenarios, from which we will deduce the boundary of acceptability. Our study represents a start, but is restricted in scope. A more comprehensive program of research will be required in order to inform researchers and ethics boards about the many different kinds of data that researchers might wish to collect. As people’s attitudes with respect to privacy issues change over time, it is likely that this research will have to be sustained.

A third approach is to give the participants the ability to turn data streams off and on. If this is made very transparent, as we advocated earlier, then participants can simply choose not to collect data that they do not wish to be available.

A fourth approach is to examine which projects users choose to participate in within a data marketplace. As with similar marketplaces, such as Mechanical Turk or Prolific Academic, the market will arrive at and continue to track the boundary of acceptability as it changes. One might anticipate that in time regulatory oversight would be required.

It seems likely that in the foreseeable future a combination of these approaches will be necessary.

### Search and visualization interfaces

For participants to actively maintain their privacy, it is critical that the system have a usable mechanism to allow participants to understand which data they are allowing researchers to access. That interface will also be critical to providing participants with the ability to delete portions of the data that they do not wish to be available. It is typically the case in experimental protocols that participants are afforded the right to have their data deleted should they wish. In practice, however, the mechanisms to allow people to sift through their data are rudimentary, so very few participants ever make a deletion request.

Making data available to participants in a usable form is the most difficult and perhaps the most underappreciated component of a privacy-preserving platform. For analysis purposes, we often store data in cryptic files or relational databases. Participants are more likely, however, to be familiar with search engines and should be provided with such a mechanism to access their data. However, search engines are only as good as the tags that are indexed to recover the data. For instance, you might wish to collect GPS data, and the coordinates alone might be sufficient for your purposes. However, they will only be usable by participants if they can be referenced by address, so additional effort would be required. Similarly, participants need straightforward interfaces in order to be able to select data by date and to visualize the data stored, in the form of maps or calendars, so they can truly understand what they are allowing researchers access to.

Beyond their usefulness when completing transactions with researchers, search and visualization interfaces can be intrinsically motivating—thus encouraging the engagement of participants with their data. A search tool provides a form of memory prosthesis that people can use to recall what they were doing at any given time. A visualization tool can allow users to discover patterns and relationships in their lives about which they may not have been conscious. These kinds of facilities are critical if we are to transform into a more data-aware populace.

### Analyzing data

The emergence of powerful re-identification algorithms demonstrates not just a flaw in a specific anonymization technique(s), but the fundamental inadequacy of the entire privacy protection paradigm based on “de-identifying” the data. De-identification provides only a weak form of privacy. It may prevent “peeping” by insiders and keep honest people honest. Unfortunately, advances in the art and science of re-identification, increasing economic incentives for potential attackers, and ready availability of personal information about millions of people (for example, in online social networks) are rapidly rendering it obsolete. (Narayanan & Shmatikov, 2010, p. 26)

If the “release and forget” approach employed by open repositories is not a viable solution to providing greater transparency in science, then what can be done? Narayanan and Shmatikov (2010) argued that a better (though not foolproof) solution is to define privacy in terms of computation rather than in terms of data. Rather than provide access to the data directly, researchers would be given an application programming interface with which to interact with the data. Although researchers would be unable to access the raw data, they would be able to run analyses that do. Code could be written that ran on the raw data and tested hypotheses, but that provided only the inferential statistics and groupwise descriptive statistics to the researcher. These measures are derived from many participants and could be constructed to bind the probability that individual data could be reconstructed from the composite measures. This notion has been formally encapsulated in the concept of differential privacy that we will discuss next.

#### Understanding differential privacy

Differential privacy is a quantifiable probabilistic definition of what it means to guarantee privacy, motivated by cryptography (Dwork, McSherry, Nissim, & Smith, 2006; Dwork & Nissim, 2004). Suppose a database consists of the body weights of 20 participants, and we wish to allow a (potentially malicious) analyst to compute the average without providing access to individual data. One might imagine that providing the mean would not be a breach of privacy, as any individual’s value could not be reconstructed uniquely from the mean. However, one must consider what can be learned about a person if the attacker has access to additional information. For example, if the attacker can issue another query to the system that includes all of the same people as before, except for the individual of interest, then the weight of that person can be readily discerned. Differential privacy prevents such inference, by reporting a noisy answer to the query. If one scales the noise appropriately, one can decrease the probability that the individual’s weight can be ascertained, while also preserving the usefulness of the data analysis platform.

Formally, let *D* and *D'* be any pair of databases that differ in exactly one entry (called “neighbors”), and the mechanism *M*(.) be a random function that takes in a database and outputs a random output such as a real vector. We call the mechanism (*ϵδ*)-differentially private if, for all neighboring databases *D* and *D'* and measurable sets *S* of the range of the output, the inequality *P*(*M*(*D*) ∈ *S*) ≤ *e*^*ϵ*^*P*(*M*(*D*′) ∈ *S*) + *δ* holds.

The interpretation of the inequality is as follows. Suppose *D* is a database of sensitive measurements from *N* participants, and *D'* is a similar database of the same participants except one particular participant, whose data are included in *D'* but not in *D*. The mechanism *M*(*D*) is the result of an analysis that the analyst has queried, such as statistics of the data *D*, plus some random noise to the answer to provide privacy. If the mechanism *M* is differentially private, then it is difficult to infer whether the output *M*(.) is from *D* or *D'* (whose probabilities differ only by the multiplicative and additive constants *ϵ* and *δ*). Furthermore, since *D* and *D'* differ by only one entry, it is difficult to guess whether a particular participant was included (*D'*) or not included (*D*) in the output, even if the adversary knows the sensitive data of the *N*–1 participants.

Differential privacy packages such as diffpriv.R^2^ demonstrate how this concept can be implemented. Although they are useful for illustrative purposes, they do not provide a solution in themselves, since to apply them one must have access to the raw data. Privacy protection is only afforded when such a package is incorporated into a data collection and access control platform.

### The experimental platform

Although some analyses can be completed using only the experience-sampling data, it is commonly necessary to also administer experimental paradigms. For instance, in an experiment we are intending to run, participants are presented with a map and are asked where they were at a given time. Four alternatives are presented, and participants make a response. Rather than have researchers examine a person’s data and select the alternatives, these are chosen by the experimental code, which is run within a password-protected environment. The participant makes selections, and the data are added to the participant’s personal repository. The researcher also has access to these records, but they contain only the event identifiers (random keys) that correspond to the target and distractor coordinates. This approach allows researchers to run subsequent analyses that incorporate factors such as the distance between points, without having access to the GPS coordinates themselves. In other experiments, we have created similar algorithms that select images for presentation, automatically excluding those that are too dark, or blurred, or that contain too little information.

Although not being able to examine the stimuli that have been presented to participants can make it more difficult to debug code and discover regularities, it also introduces a wholesome discipline in the stimulus selection process. For example, the stimuli that are chosen by researchers might be subject to subtle selection biases that might be confounded with the substantive questions under investigation. Requiring that a publishable algorithm be responsible for the selection makes the process more transparent. The algorithms can and should be published in order to make the selection process more transparent. The human selection of stimuli can be subject to subtle biases that might influence results in ways that are not communicated and of which the experimenters might not be aware—thus compromising replication and scientific understanding.

### Legal protections

As we advocated in the section entitled “Who Owns Data?,” users should retain ownership of their data and be free to license the data to multiple researchers. Examples of legal agreements that enshrine this principle can be found on the unforgettable.me website (the user agreement can be found at www.unforgettable.me/terms, the researcher agreement at www.unforgettable.me/researcher-terms, and the privacy policy at www.unforgettable.me/privacy).

Researchers should take a couple of implications of this policy into consideration:

Any data generated by experiments belong to the users. Therefore, the users retain the right to license the data to other researchers. For another researcher to be able to do that, however, the researcher must be able to find the data. On the Unforgettable.me research platform a unique key is generated for each experiment, and it is assigned to data generated by a researcher’s experimental code. This key can then be published with the corresponding article, thus avoiding scooping while also ensuring that the data will be available for others to replicate analyses.

Another consequence of the policy is that users retain the right to delete data even after analyses have been conducted. In principle, this policy is already in force in most circumstances today. However, the difficulty involved in actually accessing data means that the right is seldom exercised. If one implements more comprehensive search and visualization interfaces, data deletion will become easier, so it will likely occur more regularly. Although this aspect of the system might undermine the ability to reproduce analyses in some circumstances, it is a necessary evil if one is to genuinely implement privacy rights.

## Discussion

Privacy and open science are on a collision course. The experience-sampling techniques that promise to revolutionize the psychological sciences are also the techniques that are most invasive to privacy. Openly publishing such data is not an option. We have proposed a solution that relies on collection mechanisms that provide the user with precise control over what is collected, search and visualization mechanisms that give users tools to understand and delete their data, an analysis platform that allows researchers to conduct analyses without seeing the raw data, an experimental platform that allows users to respond to their own data without exposing them to researchers, and a legal framework that cedes ownership of data to the users.

The long-term impact of using experience-sampling data to understand psychological processes could be transformative. To reach that goal, though, we need to reinvent our relationship to data, to protect the partnership we share with our participants.

### Author note

This research was supported by the Australian Government through the Australian Research Council’s Discovery Projects funding scheme (project DP150100272), and through a Discovery Early Career Research Award (project DE170100106, awarded to A.F.O.). The views expressed herein are those of the authors and are not necessarily those of the Australian Government or the Australian Research Council. *Conflict of interest:* A critical part of the open science movement involves the open disclosure of both the explicit and potential implicit motivations for scientific work. In that spirit, the reader should be aware that S.D. is the CEO of a startup called Unforgettable Research Services Pty Ltd (URS) that specializes in providing privacy-preserving experience-sampling collection and analysis services. B.S. is the chief technical officer of URS.

## Appendix: Example privacy principles

Public institutions in Victoria, Australia, are subject to the following guidelines on the collection and use of personal and/or sensitive information (Privacy and Data Protection Act 2014).

Principle 1—Collection

1.1 An organisation must not collect personal information unless the information is necessary for one or more of its functions or activities.

1.2 An organisation must collect personal information only by lawful and fair means and not in an unreasonably intrusive way.

1.3 At or before the time (or, if that is not practicable, as soon as practicable after) an organisation collects personal information about an individual from the individual, the organisation must take reasonable steps to ensure that the individual is aware of—

(a)the identity of the organisation and how to contact it; and

(b)the fact that the individual is able to gain access to the information; and

(c)the purposes for which the information is collected; and

(d)to whom (or the types of individuals or organisations to which) the organisation usually discloses information of that kind; and

(e)any law that requires the particular information to be collected; and

(f)the main consequences (if any) for the individual if all or part of the information is not provided.

1.4 If it is reasonable and practicable to do so, an organisation must collect personal information about an individual only from that individual.

1.5 If an organisation collects personal information about an individual from someone else, it must take reasonable steps to ensure that the individual is or has been made aware of the matters listed in IPP 1.3 except to the extent that making the individual aware of the matters would pose a serious threat to the life or health of any individual.

Principle 2—Use and Disclosure

2.1 An organisation must not use or disclose personal information about an individual for a purpose (the secondary purpose) other than the primary purpose of collection unless—

(a)both of the following apply—

(i)the secondary purpose is related to the primary purpose of collection and, if the personal information is sensitive information, directly related to the primary purpose of collection;

(ii)the individual would reasonably expect the organisation to use or disclose theinformation for the secondary purpose; or

(b)the individual has consented to the use or disclosure; or

(c)if the use or disclosure is necessary for research, or the compilation or analysis of statistics, in the public interest, other than for publication in a form that identifies any particular individual

(i)it is impracticable for the organisation to seek the individual’s consent before the use or disclosure; and

(ii)in the case of disclosure—the organisation reasonably believes that the recipient of the information will not disclose the information;

Principle 4—Data Security

4.1 An organisation must take reasonable steps to protect the personal information it holds from misuse and loss and from unauthorised access, modification or disclosure.

4.2 An organisation must take reasonable steps to destroy or permanently de-identify personal information if it is no longer needed for any purpose.

Principle 5—Openness

5.1 An organisation must set out in a document clearly expressed policies on its management of personal information. The organisation must make the document available to anyone who asks for it.

5.2 On request by a person, an organisation must take reasonable steps to let the person know, generally, what sort of personal information it holds, for what purposes, and how it collects, holds, uses and discloses that information.

Principle 8—Anonymity

8.1 Wherever it is lawful and practicable, individuals must have the option of not identifying themselves when entering into transactions with an organisation.

Principle 9—Transborder Data Flows

9.1 An organisation may transfer personal information about an individual to someone (other than the organisation or the individual) who is outside Victoria only if—

(a)the organisation reasonably believes that the recipient of the information is subject to a law, binding scheme or contract which effectively upholds principles for fair handling of the information that are substantially similar to the Information Privacy Principles; or

(b)the individual consents to the transfer; or

(c)the transfer is necessary for the performance of a contract between the individual and the organisation, or for the implementation of precontractual measures taken in response to the individual’s request; or

(d)the transfer is necessary for the conclusion or performance of a contract concluded in the interest of the individual between the organisation and a third party; or

(e)all of the following apply—

(i)the transfer is for the benefit of the individual;

(ii)it is impracticable to obtain the consent of the individual to that transfer;

(iii)if it were practicable to obtain that consent, the individual would be likely to give it; or

(f)the organisation has taken reasonable steps to ensure that the information which it has transferred will not be held, used or disclosed by the recipient of the information inconsistently with the Information Privacy Principles.

## Footnotes

1 To summarize, GPS similarities were found to be the most significant predictors, followed by audio and accelerometry similarities, and then image similarities. These results were unexpected, since the images were used as the retrieval cues, so one might have expected image similarity to play an important role in the memory retrieval process.

2
cran.r-project.org/web/packages/diffpriv/index.html
